# Safety and efficacy of antimicrobial optimization based on negative results from BioFire FilmArray Pneumonia panel and respiratory culture

**DOI:** 10.1017/ash.2025.10117

**Published:** 2025-09-18

**Authors:** Noah Yoo, Xian Jie Cindy Cheng, Juri Chung, Shalinee Chawla, Ioannis Zacharioudakis, Yanina Dubrovskaya

**Affiliations:** 1 Department of Pharmacy, NYU Langone Health - Long Island, Mineola, NY, USA; 2 Department of Pulmonary Critical Care, NYU Langone Hospital - Long Island, Mineola, NY, USA; 3 Department of Infectious Disease, Department of Medicine, NYU Langone Health, New York, NY, USA; 4 Department of Pharmacy, NYU Langone Health, New York, NY, USA; 5 Department of Medicine at NYU Grossman School of Medicine, New York, NY, USA

## Abstract

**Background:**

The BioFire FilmArray Pneumonia (BFP) panel is a multiplexed nucleic acid test intended to detect respiratory pathogens from sputum or bronchoalveolar lavage (BAL) specimens. Efficacy and safety of de-escalation strategies in patients with negative BFP results remain unclear.

**Methods:**

This was a multicenter, retrospective analysis of patients with suspected pneumonia and negative BFP and respiratory cultures. Patients were stratified into two groups: those whose antibiotic therapy was discontinued or withheld within 48 hours of a negative BFP (ATDW group) and those whose antibiotic therapy was continued (ATC group). We evaluated composite primary outcome of in-hospital mortality and 30-day readmission due to pneumonia (PNA) or recurrent PNA during index admission and secondary safety outcomes.

**Results:**

Among 500 patients with negative BFP assay, a total of 185 patients were included in the final analysis (59 ATDW vs. 126 ATC). The ATDW group had significantly shorter total duration of antibiotic therapy (1 day vs 7 days, *p* < 0.001). The primary composite outcome was similar between ATDW and ATC groups (23.1% vs 35.3%, *P* = 0.15). Multivariate analysis identified ICU admission and/or intubation (OR 7.5, 95% CI 3.17–17.52, *P* < 0.001) as only independent predictor of the composite primary outcome. The ATDW group experienced fewer rates of acute kidney injury (AKI)(8% vs 37%, *P* = 0.004).

**Conclusion:**

Antimicrobial optimization based on negative results from both BFP and respiratory culture may potentially reduce unnecessary antibiotic exposure and AKI in hospitalized patients with suspected pneumonia without increasing the risk of mortality and readmission.

## Introduction

Lower respiratory tract infections (LRTIs) are the leading infectious disease cause of death in the world and the fifth overall cause of death.^1^ Hospital-acquired pneumonia (HAP) and ventilator-associated pneumonia (VAP) account for more than a fifth of hospital-acquired infections and are associated with dramatic increases of both hospital length of stay, cost of care, and mortality.^2^ Patients with LRTIs are often initiated on broad-spectrum antibiotic therapies. However, traditional culture methods can often take 72 hours or longer, which may delay the optimization and de-escalation of antibiotics and potentially increase the risk of resistance, toxicities, and unnecessary cost.^3^ Early pathogen identification in patients with pneumonia (PNA) facilitates targeted antibiotic therapy and enables timely optimization of the treatment regimen. Likewise, early confirmation of the absence of pathogen detection may facilitate the discontinuation of unnecessary antibiotics when clinically appropriate.

The BioFire FilmArray Pneumonia (BFP) panel is a multiplexed nucleic acid test intended for simultaneous detection and identification of multiple respiratory viral and bacterial nucleic acids, along with specific antimicrobial resistance markers, in sputum or bronchoalveolar lavage (BAL) specimens obtained from individuals suspected of LRTI. BFP utilizes polymerase chain reaction (PCR), a type of nucleic acid amplification test (NAAT), which amplifies specific DNA or RNA sequence to enable rapid and sensitive detection of pathogens. BFP and other rapid respiratory panels can play a crucial role in streamlining the process of antibiotic de-escalation or discontinuation and deterring resistance by reducing the use of inappropriately broad-spectrum antibiotics, given its high specificity (97.2%) and rapid turnaround time (approximately 1 h) for identifying potential pathogens.^6,7^

Previous studies have shown that rapid PCR identification methods for identification of respiratory pathogens (eg, BFP or Unyvero) have the potential to reduce unnecessary antimicrobial exposure and enhance the appropriateness of empiric antibiotic therapy in adult patients with PNA.^8–10^ Other studies have demonstrated PCR-based identification of organisms in the diagnosis and management of PNA is associated with a reduction in inappropriate use of antibiotics.^11–13^ However, these analyses have limitations, including confounding variables related to COVID-19 pneumonia, insufficient education to providers on the use of PCR-based methods, and lack of evaluation regarding the clinical impact of PCR-guided therapy.^11–13^ Studies evaluating the safety and efficacy of de-escalation based on negative PCR are limited. In this study, we aimed to evaluate the safety and clinical outcomes of antimicrobial optimization strategy in patients who tested negative on both BFP and respiratory culture.

## Methods

### Study design

This was a multicenter, retrospective cohort study of patients admitted to a large academic health system from 1/2022 to 9/2023. Patients were included if they were aged 18 years or older, had a suspicion of PNA based on clinical imaging (computed tomography [CT] of the chest or chest X-ray [CXR]) but had a respiratory sample of sputum or BAL that resulted negative on both respiratory sample and BFP. Patients were excluded if they received antibiotics for 72 hours or longer prior to BFP, had concomitant infections requiring antibiotics, had discordant BFP result, did not have a respiratory sputum or BAL cultures to correspond to BFP, was either made comfort care, died, or left against medical advice within 72 hours of BFP, or was admitted for organ transplant.

### Institutional practice/guidelines

Institutional guidance with recommendations on the use of BFP to guide antimicrobial therapy in hospitalized adult patients with LRTIs were put together by our Diagnostic and Antimicrobial Stewardship programs effective 5/2021 (Supplementary material on PNA Panel Guideline). This guidance includes criteria for BFP use, frequency of testing and recommendations on adjustment of empiric therapy based on BFP results. Considerations for the first line therapy based on detected pathogen is provided in the table format. There are no specific recommendations when BFP reports all pathogens as non-detected.

### Data collection, intervention, and definitions

This study was approved by the NYU Langone Health Institutional Review Board. A list of hospitalized patients with negative BFP test results was extracted from our electronic health records (EHR). Manual chart review of EHR was utilized to further evaluate for inclusion and collect pertinent data. The presence of immunosuppression, antibiotic allergies, clinical imaging (CT or CXR) were reviewed and documented. Charlson Comorbidity Index (CCI),^15^ quick Pitt Bacteremia Score (qPBS) ^16^ were generated for included patients.

For data regarding interventions, we collected the type of BFP sample (sputum or BAL), antibiotic type, and antibiotic duration. The duration of each antibiotic therapy was calculated as the time from the first to the last dose of that specific antibiotic and was reported in days. The total duration of antibiotic therapy was determined based on the time between the first and last administered dose of any antibiotic, regardless of changes in antibiotic type. Antibiotics were classified as anti-MRSA therapy (vancomycin, linezolid, daptomycin, or ceftaroline); antipseudomonal therapy (ceftazidime, aztreonam, cefepime, piperacillin-tazobactam, meropenem, levofloxacin, or other novel beta-lactam/beta-lactamase inhibitor combinations with activity against *Pseudomonas aeruginosa*); anti-atypical therapy (doxycycline or azithromycin); and other antibiotics used for the treatment of PNA, such as ceftriaxone and ampicillin-sulbactam.

The definition of acute kidney injury (AKI) was based on KDIGO criteria ^14^ and was assessed during the antibiotic treatment course. The 30-day readmission was defined as documented readmission within 30 days due to clinically suspected pneumonia. Recurrent PNA during index admission was defined as a case of suspected pneumonia during the same hospitalization, separate from the case BFP was initially collected for. Transaminitis was defined as aspartate transaminase (AST) and/or alanine aminotransferase (ALT) increase to or above 5 times upper limit of normal (UNL).

### Study primary and secondary outcomes

We compared the outcomes in patients who had their antibiotic therapy discontinued or withheld within 48 hours of negative BFP results (ATDW group) to those who had antibiotic therapy continued despite negative BFP results (ATC group). We evaluated a composite primary outcome of in-hospital mortality and 30-day readmission due to PNA or recurrent PNA during index admission. Secondary safety outcomes were AKI including the need for new onset dialysis during treatment, and other antibiotic-related adverse events such as transaminitis, allergic reaction, and *Clostridioides difficile* Infections (CDI) within 30 days of the first dose of antibiotics.

### Statistical analysis

Categorical data are presented as frequencies, and continuous data are presented as medians and interquartile range (IQR) for the full cohort, ATDW and ATC groups. The two groups were compared using the χ^2^ test or Fisher exact test for categorical variables and the Mann–Whitney test for continuous data. Statistical significance was defined by a 2-sided *P* < .05. A regression analysis was conducted to identify independent predictors of the composite primary outcome. The validity of the model was assessed by estimating goodness-of-fit with Hosmer–Lemeshow test (*P* = .391). All analyses were conducted with SPSS version 28 (IBM Corp, Armonk, New York).

## Results

### Patients

**Figure 1.**
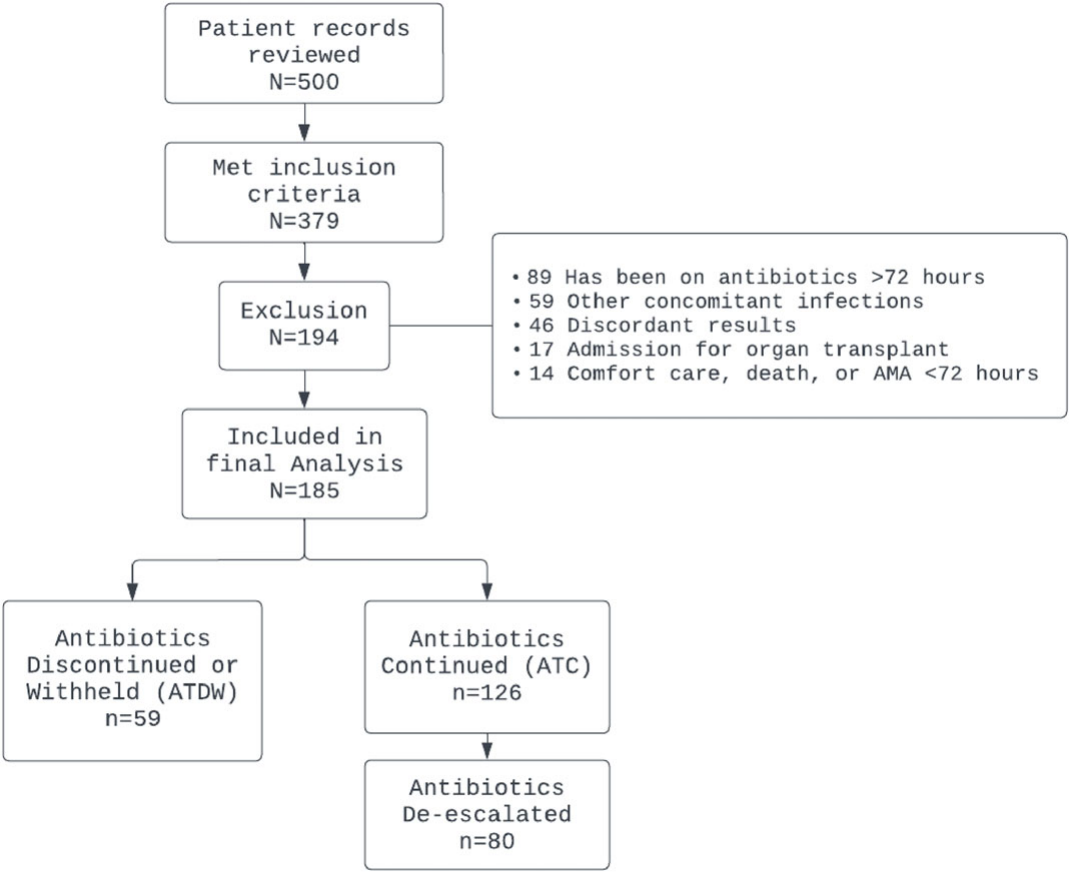
Diagram of inclusion and exclusion criteria.

A total of 500 patients were assessed for eligibility from 1/1/2022 to 9/30/2023, of which 379 patients met the inclusion criteria. A total of 185 patients were included in the final analysis (Figure 1). The most common reasons for exclusion were receiving >72 hours of antibiotics prior to BFP testing (30.16%) or concomitant infections (20.63%) requiring antibiotic therapy. Among 185 patients who were included, 59 patients (31.9%) had antibiotic therapy discontinued (*n* = 31) or not initiated (*n* = 28) within 48 hours of negative BFP result (ATDW group), while 126 patients (68.1%) had antibiotic therapy continued (ATC group) with 80 patients (63.49%) who had antibiotics de-escalated.

The baseline characteristics including the presence of immunosuppression were generally well balanced between groups (Table 1). Median age of the cohort was 68 years, and 52.9% were male. Charlson Comorbidity Index was similar between the groups (ATDW group 7 vs ATC group 8, *P* = .25). A total of 22. 2% of patients had allergies to antibiotics (penicillin (15.7%) and cephalosporins (2.7%). The hospital length of stay was similar between groups (ATDW group 8.3 d vs ATC group 7.8 d, *P* = .96). In regard to the type of pneumonia, patients in the ATC group were more likely to have CAP (59.3 vs 78. 6%, *P* = .006), while the ATDW group were more likely to have HAP (37. 3% vs 17. 5%, *P* = .003). Patients in the ATC group had higher white blood cell (WBC) count (13.2 cells/uL [7.40–18.10] vs 10.0 cells/uL [7.4–13.3] in ATDW group, *P* = .04). The rate of ICU admission was not different (ATDW 40.7% vs ATC 52. 4%, *P* = .14), whereas the duration of ICU stay was longer in the ATC group (ATDW 4.36 [1.08–9.88] vs ATC 6.93 [3.27–13.41], *P* = .045). The qPBS distribution was higher in the ATC group (1 [0–3 vs 1 [0–1] in ATDW], *P* = .02).

**Table 1.** Baseline characteristics

	Total N = 185	ATDW group n = 59	ATC group n = 126	*P*-value
Age (median, IQR)	68 (60.1–78.6)	72 (60.1–82.1)	67 (58.8–76.7)	.28
Gender Male Female	98 (53.0) 87 (47.0)	27 (45.8) 32 (54.2)	71 (56.4) 55 (43.7)	.18
Ethnicity Caucasian Black or African American Asian Hispanic Other	109 (58.9) 24 (13.0) 13 (7.0) 19 (10.3) 18 (9.7)	33 (55.9) 8 (13.6) 6 (10.2) 4 (6.8) 8 (13.6)	76 (60.3) 16 (12.7) 13 (10.3) 9 (7.1) 10 (7.9)	.57 .87 .98 1.00 .23
Past Medical History Past or current smoker Malignancy COPD Asthma CKD Home oxygen Interstitial or chronic lung disease ESRD Chronic trach Pulmonary fibrosis	65 (35.1) 58 (31.4) 42 (22.7) 21 (11.4) 26 (14.1) 32 (17.3) 15 (8.1) 12 (6.5) 2 (1.1) 2 (1.1)	20 (33.9) 16 (27.1) 15 (25.4) 10 (17.0) 9 (15.3) 6 (10.2) 5 (8.5) 4 (6.8) 1 (1.7) 1 (1.7)	45 (35.7) 42 (33.3) 27 (21.4) 11 (8.7) 17 (13.5) 26 (20.6) 10 (7.9) 8 (6.4) 1 (.8) 1 (.8)	.81 .40 .55 .10 .75 .08 1.00 1.00 .54 .54
Charlson Comorbidity Index	8 (5–10)	7 (5–10)	8 (5–10)	.25
Immunocompromised	50 (27.0)	13 (22.0)	37 (29.4)	.30
Type (N, %) Immunosuppressive medications Chemo/radiation/immunotherapy<3 month Prednisone ≥20 mg/d Immunocompromising condition ANC<500 (cells/uL)	19 (38.0) 22 (44.0) 5 (10.0) 5 (10.0) 3 (6.0)	N = 13 7 (53.9) 6 (46.2) 1 (7.7) 0 0	N = 37 12 (32.4) 16 (43.2) 4 (10.8) 5 (13.5) 3 (8.1)	
Antibiotic allergies Penicillin Cephalosporin Sulfa antibiotic Fluoroquinolone Tetracycline Aminoglycoside Macrolide Carbapenem Vancomycin Clindamycin NKA	29 (15.7) 5 (2.7) 10 (5.4) 5 (2.7) 2 (1.1) 2 (1.1) 2 (1.1) 1 (.5) 1 (.5) 1 (.5) 144 (77.8)	8 (13.6) 2 (3.4) 2 (3.4) 2 (3.4) 2 (3.4) 2 (3.4) 1 (1.7) 0 0 0 46 (78.0)	21 (16.7) 3 (2.4) 8 (6.4) 3 (2.4) 0 0 1 (.8) 1 (.8) 1 (.8) 1 (.8) 98 (77.8)	.59 .66 .51 .66 .10 .10 1.00 1.00 1.00 1.00 .98
CXR reading Interstitial opacities Consolidation Infiltrates Pulmonary cavitation Other	146 (78.9) 34 (18.4) 16 (8.7) 3 (1.6) 24 (13.0)	47 (79.7) 9 (15.3) 5 (8.5) 0 11 (18.6)	99 (78.6) 25 (19.8) 11 (8.7) 3 (2.4) 13 (10.3)	.87 .45 .95 .55 .12
Hospital length of stay (median, IQR)	8.0 (5.0–14.2)	8.3 (4.4–14.1)	7.8 (5.1–15.7)	.96
Pneumonia type CAP HAP VAP	134 (72.4) 44 (23.8) 7 (3.8)	35 (59.3) 22 (37.329) 2 (3.4)	99 (78.6) 22 (17.5) 5 (4.0)	.006 .003 1.00
Viral PNA	15 (8.1)	6 (10.2)	9 (7.1)	.57
Covid PNA	24 (13.0)	11 (18.6)	13 (10.3)	.12
Prior hospitalization<90 d	72 (38.9)	21 (35.6)	51 (40.5)	.53
Highest WBC within 48 h	11.7 (7.4–16.5)	10.0 (7.4–13.3)	13.2 (7.4–18.1)	.04
qPitt Bacteremia Score	1 (0–2)	1 (0–1)	1 (0–3)	.02
ICU admission and/or intubation	93 (50.3)	24 (40.7)	69 (54.8)	.10
ICU admission	90 (48.7)	24 (40.7)	66 (52.4)	.14
ICU Type MICU NICU SICU CCU	61 (33.0) 9 (4.9) 11 (6.0) 8 (4.3)	N = 24 11 (18.6) 6 (10.2) 4 (6.8) 2 (3.4)	N = 66 50 (39.7) 3 (2.4) 7 (5.6) 6 (4.8)	
ICU length of stay, median (IQR)	5.8 (2.9–12.9)	4.4 (1.1–9.9)	6.9 (3.3–13.4)	.05
Intubation	63 (34.1)	17 (28.8)	46 (36.5)	.34
Duration of intubation, median (IQR)	5.0 (2.0–10.9)	4.5 (1.4–13.2)	5.0 (2.0–10.4)	.97

All data are represented as n (%) unless specified otherwise.

### Interventions

Table 2 displays comparisons of interventions. In both groups, BFP was more frequently obtained from sputum samples (67. 6%) than from BAL samples (32.4%). The ATDW group had significantly lower use of antibiotics (anti-MRSA, antipseudomonal, Atypical, ceftriaxone, and other antibiotics). Time to collection of BFP from development of PNA was 1.2 days (.6–2.0) and were not different between the two groups. The BFP turnaround time was .8 days (.5–1.0), while sputum or BAL culture turnaround time was 2.5 days (2.0–2.8). MRSA screening was performed in 75.68% of patients, and 111/140 patients had screening by culture in line with our local policy. More patients in the ATC group were screened for atypical organisms (74.6 vs 88.1%, *P* = .02) and had blood cultures collected (66.1 vs 83. 8%, *P* < .001). Among patients who were continued on antibiotics, 80 (63. 5%) had de-escalation within 48 hours. Most of this de-escalation was anti-MRSA therapy (67.5%), followed by anti-atypical therapy (50.0%). The total duration of antibiotics was shorter in ATDW group (1 vs 7 d, *P* < .001).

**Table 2.** Interventions

	Total N = 185	ATDW group n = 59	ATC group n = 126	*P*-value
BFP sample Sputum BAL	125 (67.6) 60 (32.4)	41 (69.5) 18 (30.5)	84 (66.7) 42 (33.3)	.70
Antibiotic use type Anti-MRSA Antipseudomonal Anti-atypical Ampicillin-sulbactam Ceftriaxone Other	101 (54.6) 119 (64.3) 94 (50.8) 28 (15.1) 47 (25.4) 18 (9.7)	17 (28.8) 21 (35.6) 16 (27.1) 6 (10.2) 9 (15.3) 0	84 (66.7) 98 (77.8) 78 (61.9) 22 (17.5) 38 (30.2) 18 (14.3)	<.001 <.001 <.001 .20 .03 .002
Duration of antibiotics, median (IQR), days Anti-MRSA Antipseudomonal Anti-atypical Ampicillin-sulbactam Ceftriaxone Other Total		2 (1..95–2.659) 3 (.765–3.325) 2 (1.10–3.02.95) 1 (.325–2.877) 1 (1.00–2.548) NA 1 (.00–2.988)	2 (1.549–4.215) 6 (3.81–9.23) 3 (2.01.95–5.04.97) 2 (.91–4.03.96) 4 (2.106–6.05.95) 2 (1.545–4.60) 7 (4.765–8.989)	.042 <.001 .0545 .1875 .001 NA <.001
Time to PCR from admission, median (days)	1.8 (.9–3.1)	2.3 (1.1–5.2)	1.6 (.7–2.8)	<.001
Time to PCR from development of PNA, median (days)	1.2 (.6–2.0)	1.3 (.8–2.3)	1.1 (.5–1.9)	.19
PCR turnaround time, median (days)	.8 (.5–1.0)	.9 (.6–1.2)	.8 (.4–1.0)	.02
MRSA screening^1^	140 (75.7)	43 (72.9)	97 (77.0)	.54
Atypical Antigen Screening	155 (83.8)	44 (74.6)	111 (88.1)	.02
Blood Culture Collected	155 (83.8)	39 (66.1)	116 (92.1)	<.001
Sputum/BAL culture turnaround time, mean (days)	2.5 (2.0–2.8)	2.5 (2.0–2.9)	2.5 (2.0–2.8)	.61
De-escalation	NA	NA	80 (63.5)	NA
De-escalation type Anti-MRSA Antipseudomonal Anti-atypical Other	NA	NA	N = 80 54 (67.5) 20 (25.0) 40 (50.0) 8 (10.0)	NA

All data are represented as n (%) unless specified otherwise.

1 MRSA/MSSA nasal screening is done by culture with turnaround time of 36h and used to measure transmission and identify individuals at greater risk for invasive infections; 29/140 tests done by PCR

### Primary and secondary outcomes

Table 3 displays comparisons of outcomes. Overall, the composite primary outcome occurred in 23.1% in ATDW group and 35.3% in ATC Group, *P* = .15. As for individual components, patients in ATDW group had a lower likelihood of in-hospital mortality (8.5 vs 20.6%, *P* = .04) whereas 30-day readmission due to PNA or PNA recurrence during index admission were similar between the groups (17.0 in ATDW vs 15.9% in ATC, *P* = .85).

**Table 3.** Primary and secondary outcomes

	ATDW group n = 59	ATC group n = 126	*P*-value
Composite primary outcome	12 (23.1)	47 (35.3)	.15
In-hospital mortality (n, %)	5 (8.5)	26 (20.6)	.04
30-day readmission for PNA or recurrent pneumonia during index admission	10 (17.0)	20 (15.9)	.85
Secondary Safety Outcomes			
Incidence of AKI (n, %)	5 (8.5)	34 (37.0)	.004
New onset dialysis (n, %)	0	4 (3.2)	.31
Allergic reaction (n, %)	1 (1.7)	3 (2.4)	1.00
Incidence of transaminitis (n, %)	4 (6.8)	8 (6.4)	1.00
Incidence of CDI	1 (1.7)	0	.32

All data are represented as n (%) unless specified otherwise

For secondary safety outcomes, more patients in the ATC group experienced AKI (8.5 vs 37.0%, *P* = .004). Four patients in the ATC group had an incidence of new onset dialysis (3.2 %) compared to none in the ATDC group. In the subgroup of patients who experienced AKI, the most frequent antibiotics used were vancomycin (56.41%) and piperacillin-tazobactam (56.41%) with no significant differences between groups. (Supplementary material Table 1)

The incidence of allergic reaction, transaminitis, and CDI did not differ between the groups (Table 3).

Multivariate regression analysis (Table 4) identified an ICU admission and/or intubation (OR 7.5, 95% CI 3.17–17.52, *P* < .001) as the only independent predictors of the composite primary outcome after controlling for clinical variables of interest and ATDW group (OR .6, 95% CI .26–1.15, *P* = .37).

**Table 4.** Variables associated with composite primary outcomes

			Univariate Analysis		Multivariate Analysis	
	**Met composite primary outcome n = 52**	**Did not meet composite primary outcome n = 133**	***P*-value**	**OR (95% CI)**	***P*-value**	**OR (95% CI)**
ATDW group	12 (23.1)	47 (35.3)	.15	.6 (.26–1.15)	.37	.7 (.27–1.63)
Age	68 (59–79)	68 (60–79)	.85	1.0 (.98–1.02)	.57	1.0 (.98–1.03)
Male sex	26 (50)	72 (54.1)	.73	.8 (.44–1.61)	.83	.9 (.44–1.94)
PNA Type: HAP or VAP	21 (40.4)	30 (22.6)	.04	2.3 (1.71–4.62)	.41	1.7 (.78–3.92)
CCI	8 (5–11)	8 (5–10)	.84	.99 (.90–1.09)	.43	.96 (.87–1.06)
WBC>12 or<4 10^3^/uL	33 (63.5)	65 (48.9)	.11	1.8 (.94–3.51)	.57	1.3(.57–2.76)
qPitt Bacteremia Score	1 (1–3)	1 (1–2)	.32	1.1 (.88–1.49)	.13	1.3 (.93–1.73)
ICU admission and/or intubation	43 (82.7)	50 (37.6)	<.001	7.9 (3.57–17.64)	<.001	7.5 (3.17–17.52)

## Discussion

In this multicenter, retrospective cohort study, we described antimicrobial optimization based on negative results from BFP and respiratory culture. In our selected cohort of patients, we found that withholding or discontinuing antibiotics based on the negative results of both BFP and respiratory samples when clinically appropriate was not associated with increased in-hospital mortality, 30-day readmission due to PNA or recurrent PNA during index admission. Although the baseline characteristics were comparable between groups, there were a few clinical differences. WBC within 48 hours of antibiotic initiation was higher in the ATC group. Patients in the ATDW group were also more likely to have HAP, whereas patients in the ATC group were more likely to have CAP. Although the patients included in this study had similar baseline severity of illness evident from CCI, rates of ICU admission, and the rates of intubation at the time of inclusion, the distribution of qPBS favored the ATDW group ICU admission and/or intubation were identified as the only independent predictor of our composite primary outcome in multivariate regression analysis.

BFP includes 18 different bacterial species, including 3 atypical bacteria, and 8 different viruses (9 for BFP plus), and 7 different antimicrobial resistance genes.^4^ BFP demonstrated high specificity for both sputum and BAL samples (>91%),^7^ with negative predictive value (NPV) ranging from 99.04–99.96% for common bacterial pathogens, when compared to the standard of care culturing method.^19^ Although cultures remain as the gold standard in the identification of bacterial respiratory tract pathogens, it may be difficult to accurately recover all pathogens in clinical samples due to multiple reasons—organisms being in a complex matrix, host immune response, and prior antibiotic usage.^7^ Furthermore, cultures are subject to laboratory interpretation by technicians examining cultures.^7^ On the other hand, the potential drawback of molecular methods is the detection of nonviable organisms and therefore positive results should be examined against clinical significance.^7,19^ Given these characteristics, an absence of organism detection on both BFP and respiratory culture, can aid in antibiotic de-escalation and discontinuation when clinically appropriate.

Several studies have shown the potential for PCR-based methods to optimize antimicrobial therapy. In a randomized clinical trial of 208 patients, patients who were assigned to PCR-based molecular detection (Unyvero) had significantly shorter duration of inappropriate antibiotic therapy compared to patients assigned to traditional culture methods (47.1 h in PCR group vs 85.7 h in traditional culture method group).^11^ Similarly, theoretical and simulation analyses have shown that PCR-based respiratory pathogen detection methods (BFP and Unyvero) have opportunities for de-escalation and potential antibiotic modifications, while reducing the duration of broad-spectrum antibiotic therapy.^8–10,22^ Two quasi-experimental studies showed feasibility of PCR-guided therapy implementation in the practical settings to reduce antimicrobial therapy duration in critically ill patients.^12,13^ Negative BFP and respiratory culture results in our study led to antimicrobial de-escalation, discontinuation or not initiating antibiotics if clinically appropriate. Our study also adds additional finding of lower antibiotic-associated adverse events in ATDW group.

There is limited data evaluating the clinical outcomes of PCR-based antimicrobial therapies. In the previously mentioned randomized trial by Darie et al.^11^ the authors did not find significant differences in clinical stability, length of stay, or mortality rates, although the duration of inappropriate antibiotics was significantly shorter in the PCR-based group. However, this study was limited to g organism infections, and the PCR samples were limited to BAL. Furthermore, resistance markers were also excluded in the PCR-based recommendations. Our study used a different PCR assay (BioFire Pneumonia) and included both sputum and BAL samples. Our results are consistent in that the reducing of antibiotic exposure was not associated with an increased signal for harm (length of stay, mortality, and readmission rates) in the targeted cohort of patients selected for our study who had no pathogens detected on both BFP and respiratory culture.

Our study has several limitations that need to be mentioned. Due to the nature of the study design, it is subjective to inherent retrospective bias. Patients in the ATC group potentially had higher severity of illness, which could have deterred clinicians from de-escalating or discontinuing antibiotics sooner. We tried to limit confounders by excluding patients who had other suspected infections (UTIs, SSTIs, etc.) and who had discordant results (MRSA PCR, atypical PCR, and blood cultures, etc.). All patients were required to have either sputum or BAL culture, with a suspicion of PNA on imaging. We conducted a regression analysis to identify predictors of our composite primary outcome. Nevertheless, the results of the study should be carefully interpreted due to the potential for residual confounding.

It is also possible that BFP would result in false-negative results, especially if the patient was receiving antibiotics for an extended period or if an organism is not part of the BFP panel. Non-detection or reduced sensitivity for *Klebsiella aerogenes* have previously been reported.^20,21^ To minimize the rate of false negativity all patients who received more than 72 hours of antibiotics prior to BFP were excluded, and our results show that BFP samples were collected soon after development of PNA (median 1.2 d) and excluded patients who had positive cultures and PCRs. Although this approach strengthens the identification of true-negative BFP cases, it may also limit the ability to assess the real-time clinical utility of BFP’s rapid turnaround time. Specifically, by requiring culture confirmation, the benefit of early diagnostic information was not fully reflected in our outcome analysis. Lastly, we did not evaluate the clinical outcomes of patients who had antibiotics de-escalated to a narrower spectrum in the absence of resistant organisms, rather than discontinuing antibiotics. We saw that in the ATC group, 67.5% of anti-MRSA therapies, 25% of antipseudomonal therapy, and 50% of anti-atypical therapies were discontinued within 48 hours of PCR results. Further studies should evaluate the clinical impact of PCR-based de-escalation therapy rather than complete discontinuation of antimicrobials in patients with suspected PNA.

## Conclusion

In conclusion, our study provides evidence supporting the clinical safety and efficacy of the BFP-guided antibiotic stewardship in hospitalized patients with suspected PNA. Antibiotic discontinuation or withdrawal of initiation based on negative BFP was associated with significant reduction in the risk of antibiotic-associated adverse events, without increased mortality or readmission rates. These findings underscore the potential of rapid molecular diagnostics, such as BFP, in optimizing antimicrobial stewardship practices and reducing unnecessary antibiotic exposure.

## Supporting information

10.1017/ash.2025.10117.sm001 Yoo et al. supplementary material 1 Yoo et al. supplementary material

10.1017/ash.2025.10117.sm002 Yoo et al. supplementary material 2 Yoo et al. supplementary material

## Data Availability

The data sets used and/or analyzed during the current study are available from the corresponding author on reasonable request.

## Supplementary material

The supplementary material for this article can be found at https://doi.org/10.1017/ash.2025.10117.

## References

[1] Feldman C , Shaddock E. Epidemiology of lower respiratory tract infections in adults. Expert Rev Respir Med 2019;13:63–77 30518278 10.1080/17476348.2019.1555040

[2] Barbier F , Andremont A , Wolff M , Bouadma L. Hospital-acquired pneumonia and ventilator-associated pneumonia: recent advances in epidemiology and management. Curr Opin Pulm Med 2013;19:216–228 23524477 10.1097/MCP.0b013e32835f27be

[3] Doron S , Davidson LE. Antimicrobial stewardship. Mayo Clin Proc 2011;86:1113–1123 22033257 10.4065/mcp.2011.0358 PMC3203003

[4] BioFire Diagnostics. FilmArray Pneumonia Panel: Instructions for Use. Salt Lake City, UT: BioFire Diagnostics. 2019. Available from: https://www.online-ifu.com/ITI0075

[5] BioFire Diagnostics. FilmArray Pneumonia Panel Plus: Instruction Booklet. Salt Lake City, UT: BioFire Diagnostics. 2019. Document RFIT-ASY-0142 https://www.biomerieux.com/content/dam/biomerieux-com/service-support/support-documents/instructions-for-use-and-manuals/RFIT-PRT-0895-OUS-FilmArray-Pneumoplus-Instructions-for-Use-EN.pdf

[6] BioFire Diagnostics. FilmArray Pneumonia Panel: Syndromic Infectious Disease Testing for Pneumonia. Available from: https://www.biofiredx.com/products/the-filmarray-panels/filmarray-pneumonia/ Accessed July 1, 2025

[7] Murphy CN , Fowler R , Balada-Llasat JM et al. Multicenter evaluation of the biofire filmarray Pneumonia/Pneumonia plus panel for detection and quantification of agents of lower respiratory tract infection. J Clin Microbiol 2020;58:e00128–20. 32350043 10.1128/JCM.00128-20 PMC7315029

[8] Monard C , Pehlivan J , Auger G , et al. Multicenter evaluation of a syndromic rapid multiplex PCR test for early adaptation of antimicrobial therapy in adult patients with pneumonia. Crit Care 2020;24:434 32665030 10.1186/s13054-020-03114-y PMC7359443

[9] Peiffer-Smadja N , Bouadma L , Mathy V et al. Performance and impact of a multiplex PCR in ICU patients with ventilator-associated pneumonia or ventilated hospital-acquired pneumonia. Crit Care 2020;24:366 32560662 10.1186/s13054-020-03067-2 PMC7303941

[10] Erich BJ , Kilic A , Palavecino E , et al. Evaluation of the potential impact of a multiplex rapid diagnostic panel in critically ill patients with hospital-acquired Pneumonia. Cureus 2022;14:e21716. 35251792 10.7759/cureus.21716 PMC8887693

[11] Darie AM , Khanna N , Jahn K et al. Fast multiplex bacterial PCR of bronchoalveolar lavage for antibiotic stewardship in hospitalized patients with Pneumonia at risk of gram-negative bacterial infection (Flagship II): a multicentre, randomized controlled trial. Lancet Respir Med 2022;10:877–887 35617987 10.1016/S2213-2600(22)00086-8

[12] Esplund JN , Taylor AD , Stone TJ , et al. Clinical impact of a multiplex rapid diagnostic pneumonia panel in critically ill patients. Antimicrob Steward Health Epidemiol 2023;3:e5. 10.1017/ash.2022.358 PMC9879924 36714280

[13] Miller M et al. Implementation of a rapid multiplex PCR Pneumonia panel and subsequent antibiotic de-escalation. Open Forum Infect Dis 2023;10(8):ofad382. doi: 10.1093/ofid/ofad382 37564742 PMC10411041

[14] Kellum JA et al. Kidney Disease: Improving Global Outcomes (KDIGO) Acute Kidney Injury Work Group KDIGO Clinical Practice Guideline for Acute Kidney Injury. Kidney Int Suppl 2012;2:1–138

[15] Charlson ME , Pompei P , Ales KL , MacKenzie CR. A new method of classifying prognostic comorbidity in longitudinal studies: development and validation. J Chronic Dis 1987;40:373–383. 3558716 10.1016/0021-9681(87)90171-8

[16] Henderson H , Luterbach CL , Cober E , et al. The Pitt Bacteremia Score Predicts Mortality in Nonbacteremic Infections. Clin Infect Dis 2020;70:1826–1833 31219148 10.1093/cid/ciz528 PMC7156778

[17] LiverTox: Clinical and Research Information on Drug-Induced Liver Injury [Internet]. Bethesda (MD): National Institute of Diabetes and Digestive and Kidney Diseases; 2012-. Severity Grading In Drug Induced Liver Injury. 2019. Available from: https://www.ncbi.nlm.nih.gov/books/NBK548241/ 31643566

[18] BioFire Diagnostics. FilmArray pneumonia panel instructions for use. 2019. https://www.online-ifu.com/ITI0075

[19] Ginocchio CC , Garcia-Mondragon C , Mauerhofer B , Rindlisbacher C. The EME Evaluation Program Collaborative. Multinational evaluation of the BioFire® FilmArray® Pneumonia plus Panel as compared to standard of care testing. Eur J Clin Microbiol Infect Dis 2021;40:1609–1622. 33655440 10.1007/s10096-021-04195-5 PMC7924818

[20] Yoo IY , Huh K , Shim HJ et al. Evaluation of the bioFire filmArray Pneumonia panel for rapid detection of respiratory bacterial pathogens and antibiotic resistance genes in sputum and endotracheal aspirate specimens. Int J Infect Dis 2020;95:326–331 32179139 10.1016/j.ijid.2020.03.024

[21] Nicolau-Guillaumet N , Dortet L , Jacquemin A , Mourvillier B , Muggeo A , Guillard T. Lack of detection of Klebsiella aerogenes sub-species in lung infection by the BioFire® FilmArray® Pneumonia Panel plus. Clin Microbiol Infect 2024;30:688–690 38368943 10.1016/j.cmi.2024.02.008

[22] Guillotin F , Poulain C , Gaborit B , et al. Potential impact of rapid multiplex PCR on antimicrobial therapy guidance for ventilated hospital-acquired pneumonia in critically ill patients, a prospective observational clinical and economic study. Front Cell Infect Microbiol. 2022;12:804611 35493730 10.3389/fcimb.2022.804611 PMC9043525

