# Supplementary material for: Safety and efficacy of antimicrobial optimization based on negative results from BioFire FilmArray Pneumonia panel and respiratory culture
Source: Antimicrob Steward Healthc Epidemiol. 2025 Sep 18;5(1):e226. doi: 10.1017/ash.2025.10117 (PMC12451807; doi:10.1017/ash.2025.10117)
Supplement: Yoo et al. supplementary material 1 — Yoo et al. supplementary material [file S2732494X25101174sup001.docx]

This document provides evidence-based guidance on the use of the BioFire® FilmArray® Pneumonia Panel to guide antimicrobial therapy for lower respiratory tract infections. This panel should only be considered in patients with a clinical syndrome highly suggestive of pneumonia. Patients can be colonized with organisms that are detected by the panel even when pneumonia is not present. Therefore, a positive result does not mean the patient has pneumonia or that antibiotics should be started. *Clinical judgment should be used at all times, and management decisions should NOT be based solely on BioFire® FilmArray® Pneumonia Panel results. Consider consulting Infectious Diseases for patient-specific management questions.*

**Intended Use:**

- The BioFire® FilmArray® Pneumonia Panel is an FDA-cleared multiplex PCR assay for use on **sputum** and **bronchoalveolar lavage** **(BAL)** samples with a quick turnaround time. It can detect 15 bacterial, 3 atypical bacterial, and 8 viral pathogens, as well as antimicrobial resistance genes.
- A review indicated opportunities for optimization of antimicrobial therapy by changing antimicrobials to include pathogens not previously covered, but also stopping unnecessary antimicrobials and de-escalating to narrower spectrum antibiotics.
- A decrease of unnecessary broad and extended courses of empiric antimicrobials may decrease antimicrobial side effects, development of antimicrobial resistance, and incidence of *C. difficile* infections.
- All specimens submitted for the BioFire® FilmArray® Pneumonia Panel will be simultaneously cultured regardless of clinicians’ orders. Submitted sputum samples must meet standard quality of criteria of containing less than 10 epithelial cells per low power field in direct microscopy.

**Table 1. Targets of the BioFire® FilmArray® Pneumonia Panel**

| **Gram Positive Organisms**: *Staphylococcus aureus, Streptococcus pneumoniae, Streptococcus agalactiae, Streptococcus pyogenes*   - **Resistance Genes (*Staph aureus* only):** mecA/C, MREJ |
| --- |
| **Gram Negative Organisms:** *Acinetobacter calcoaceticus-baumannii* complex, *Enterobacter cloacae* complex, *Escherichia coli, Haemophilus influenzae, Klebsiella aerogenes, Klebsiella oxytoca, Klebsiella pneumonia, Moraxella catarrhalis, Proteus* spp., *Pseudomonas aeruginosa, Serratia marcescens*   - **Resistance Genes (All Gram Negatives):** CTX-M, IMP, KPC, NDM, VIM, OXA-48-like |
| **Atypical Pathogens:** *Chlamydia pneumoniae, Legionella pneumophila, Mycoplasma pneumoniae* |
| **Viral Pathogens:** *Adenovirus, Coronavirus (excluding SARS-CoV-2), Human Metapneumovirus, Rhinovirus/Enterovirus, Influenza A, Influenza B, Parainfluenza, RSV* |

**Criteria for Use:**

1. **Radiographic findings highly suggestive of pneumonia (**airspace opacity, lobar consolidation or interstitial opacities that are new or worse compared to baseline**) with expectorated or induced sputum, tracheal aspirate or BAL sample PLUS ≥2 of the following criteria:**
   1. Reported or documented fever ≥101° Fahrenheit within last 24 hours
   2. New or worsening productive cough OR increase in respiratory sections in intubated patients
   3. Pleuritic chest pain
   4. New or worsening dyspnea or hypoxia, defined as documented blood oxygen saturation < 92%
2. **If patient meets above criteria, and is diagnosed with community-acquired pneumonia (CAP), the panel can be considered ONLY if patient meets one of the following criteria:**
   1. Immunocompromised
   2. Severe pneumonia as defined in **table 2** below
   3. Receipt of treatment with anti-MRSA or anti-pseudomonas agent
   4. Documented history of MRSA or *Pseudomonas* infection
   5. At risk for infection with multi-drug resistant organisms, defined by hospitalization within the past 90 days **with** receipt of intravenous antibiotics

**Table 2. 2007 IDSA/ATS Criteria for Defining Severe Community-Acquired Pneumonia: ≥1 major criterion or ≥3 minor criterion**

| **Major Criteria:**   - Septic shock with need for vasopressors | - Respiratory failure requiring mechanical ventilation |
| --- | --- |
| **Minor Criteria:**   - Respiratory rate ≥ 30 breaths/min - Pa_O2_/Fi_O2_ ratio ≤ 250 - Multilobar infiltrates - Confusion/disorientation - Uremia (blood urea nitrogen level ≥ 20 mg/dL) | - Leukopenia (WBC < 4,000 cells/mcL) - Thrombocytopenia (Platelets < 100,000/mcL) - Hypothermia (core temperature < 36°C) - Hypotension requiring aggressive fluid resuscitation |

**Frequency of testing:**

- BioFire® FilmArray® Pneumonia Panel should not be repeated within **7 days** of prior testing
- Changes in BioFire® FilmArray® Pneumonia Panel results are intended to provide supportive information for use with clinical judgment, clinical condition, and culture results to make decisions on antimicrobial adequacy and duration.

**Table 3. Guidance of Adjustment of Empiric Therapy Based on BioFire® FilmArray® Pneumonia Panel Results:**

- For Gram-negative Resistance Genes including IMP, KPC, NDM, OXA-48 like, VIM: please consult Infectious Disease
- If pathogen isolated in sputum culture, de-escalate to most narrow antimicrobial option once susceptibility results return
- For patients with penicillin or cephalosporin allergy, considering consulting ASP or ID for recommendations

| **Result** | **Considerations for First Line Therapy** | **Additional Comments** |
| --- | --- | --- |
| **Bacteria** | | |
| *Acinetobacter calcoaceticus-baumannii complex* | Ampicillin-sulbactam | + Amikacin (if critically ill) |
| *Chlamydia pneumoniae* | Azithromycin |  |
| *Enterobacter cloacae*  *Klebsiella aerogenes*  *Proteus spp.*  *Serratia marcescens* | Ceftriaxone | Consider *initiating* cefepime or meropenem in patients with history of MDRO. In critically ill and/or decompensating patients, consider *escalation* to meropenem. |
| *Escherichia coli*  CTX-M negative  CTX-M positive | Ceftriaxone  Meropenem |  |
| *Haemophilus influenzae* | Ceftriaxone |  |
| *Klebsiella oxytoca* | Ceftriaxone |  |
| *Klebsiella pneumonia* | Ceftriaxone |  |
| *Legionella pneumophila* | Azithromycin | Consider levofloxacin if patient critically ill or not improving on macrolide therapy |
| *Moraxella catarrhalis* | Ampicillin-sulbactam |  |
| *Mycoplasma pneumoniae* | Azithromycin |  |
| *Pseudomonas aeruginosa* | Piperacillin-tazobactam |  |
| *Staphylococcus aureus –* MecA/C negative | Oxacillin or Cefazolin | A negative panel for *S. aureus* rules out MRSA pneumonia. Consider stopping anti-MRSA antibiotics. |
| *Staphylococcus aureus –* MecA/C positive | Vancomycin |  |
| *Streptococcus agalactiae* | Ampicillin |  |
| *Streptococcus pneumoniae*  No concern for CNS infection  Coverage for CNS infection | Ampicillin  Ceftriaxone + Vancomycin |  |
| *Streptococcus pyogenes* | Ampicillin |  |
| **Viruses** | | |
| *Adenovirus*  *Coronavirus (non-SARS-CoV-2)*  *Human metapneumovirus*  *Human rhinovirus/enterovirus*  *Parainfluenza virus*  *Respiratory syncytial virus* | Symptomatic Therapy | *Adenovirus, Parainfluenza Virus, Respiratory syncytial virus*: If critically ill or immunocompromised consult Infectious Disease |
| *Influenza A, Influenza B* | Oseltamivir |  |

*MDRO: multidrug resistant organism; MRSA: methicillin resistant *Staphylococcus aureus*; CNS: central nervous system

Please contact the Antimicrobial Stewardship Program (ASP) at **212-263-1169** (Tisch/Kimmel campus) or **646-618-1685** (Brooklyn campus) for questions.

**Primary Guideline Author:**

Ioannis Zacharioudakis, MD

**Secondary Guideline Authors**:

Kassandra Marsh, PharmD

Yanina Dubrovskaya, PharmD

Vinh Pham, MD

Antimicrobial Stewardship Team

**APPROVALS:**

**ANTIMICROBIAL SUBCOMMITTEE: 03/30/2021**

**P&T APPROVAL DATE: 04/08/2021**

**MEDICAL BOARD APPROVAL DATE: 05/05/2021**
