# Supplementary material for: Safety and efficacy of antimicrobial optimization based on negative results from BioFire FilmArray Pneumonia panel and respiratory culture
Source: Antimicrob Steward Healthc Epidemiol. 2025 Sep 18;5(1):e226. doi: 10.1017/ash.2025.10117 (PMC12451807; doi:10.1017/ash.2025.10117)
Supplement: Yoo et al. supplementary material 2 — Yoo et al. supplementary material [file S2732494X25101174sup002.docx]

**Supplementary Material**

**Table 1. Frequencies of antibiotics in Subgroup of patients experiencing acute kidney injury**

|  | **Total**  **N = 39** | **Total, %** | **ATDW**  **n = 5** | **ATDW, %** | **ATC**  **n = 34** | **ATC, %** | **P = value** |
| --- | --- | --- | --- | --- | --- | --- | --- |
| Piperacillin-  tazobactam | 22 | 56.4% | 2 | 40% | 20 | 58.8% | 0.64 |
| Vancomycin | 22 | 56.4% | 2 | 40% | 20 | 58.8% | 0.64 |
| Meropenem | 6 | 15.4% | 0 | 0 | 6 | 17.7% | 0.57 |
| TMP-SMX | 5 | 12.8% | 2 | 40% | 3 | 8.8% | 0.11 |
| Aminoglycoside | 3 | 7.7% | 1 | 20% | 2 | 5.9% | 0.35 |
| Cefepime | 3 | 7.7% | 1 | 20% | 2 | 5.9% | 0.35 |
| Ampicillin- sulbactam | 2 | 5.1% | 0 | 0 | 2 | 5.9% | 1 |
| Other | 19 | 48.7% | 2 | 40% | 17 | 50.0% | 1 |
